# Traumatic subclavian arterial rupture: a case report and review of literature

**DOI:** 10.1186/1749-7922-7-18

**Published:** 2012-06-18

**Authors:** Marco Assenza, Leonardo Centonze, Lorenzo Valesini, Gabriele Campana, Mario Corona, Claudio Modini

**Affiliations:** 1 Emergency Department, Division of Emergency Surgery and Trauma, Policlinico “Umberto I”, Rome, Italy; 2 Department of Radiological Sciences, Vascular and Interventional Radiology Unit, Policlinico “Umberto I”, Rome, Italy; 3 Umberto I General Hospital, University of Rome “Sapienza”, Rome, Italy; 4 Surgical Research Fellow, Via Demetriade 58, Rome, 00178, Italy

**Keywords:** Subclavian arterial rupture, Blunt chest trauma, Endovascular stent grafting

## Abstract

Subclavian artery injuries represent an uncommon complication of blunt chest trauma, this structure being protected by subclavius muscle, the clavicle, the first rib, and the deep cervical fascia as well as the costo-coracoid ligament, a clavi-coraco-axillary fascia portion. Subclavian artery injury appears early after trauma, and arterial rupture may cause life-treatening haemorrages, pseudo-aneurysm formation and compression of brachial plexus. These clinical eveniences must be carefully worked out by accurate physical examination of the upper limb: skin color, temperature, sensation as well as radial pulse and hand motility represent the key points of physical examination in this setting. The presence of large hematomas and pulsatile palpable mass in supraclavicular region should raise the suspicion of serious vascular injury. Since the first reports of endovascular treatment for traumatic vascular injuries in the 90’s, an increasing number of vascular lesions have been treated this way. We report a case of traumatic subclavian arterial rupture after blunt chest trauma due to a 4 meters fall, treated by endovascular stent grafting, providing a complete review of the past twenty years’ literature.

## Introduction

Traumatic subclavian arterial rupture represents an uncommon complication of blunt chest trauma. The subclavian artery is protected by subclavius muscle, the clavicle, the first rib, and the deep cervical fascia, as well as the costo-coracoid ligament, a clavi-coraco-axillary fascia portion. Clavicular Fractures were cited as the cause of 50% of traumatic subclavian artery injuries [1]. Arterial rupture usually causes life-threatening haemorragies, and must be carefully ruled out by physical examination as well as diagnostic imaging. Physical examination of the upper limb must focus on skin color, temperature, sensation, hand motility well as radial pulse [2]. Contrast-CT represents a key diagnostic exam, while arteriography offers both a diagnostic a therapeutic approach.

Open surgery represents the classical management of subclavian rupture, but it is associated with high morbidity mostly because the need of extensive incisions, which require lengthy healing and rehabilitation.

In recent years endovascular stent grafting, thank to technical evolution and growing operators’ experience, has become an attractive therapeutic approach to such kind of injuries, provided with less invasiveness and morbidity [3].

We report a case of traumatic subclavian arterial rupture after blunt chest trauma and clavicular fracture due to a 4 meters fall, treated by endovascular stent grafting.

## Case report

A previously healthy 70-year old man had a fall from a 4 meters high scaffold: he reported a blunt chest trauma and a cranial trauma with temporary loss of consciousness. Immediately after trauma he was brought to our hospital.

**Figure 1:**
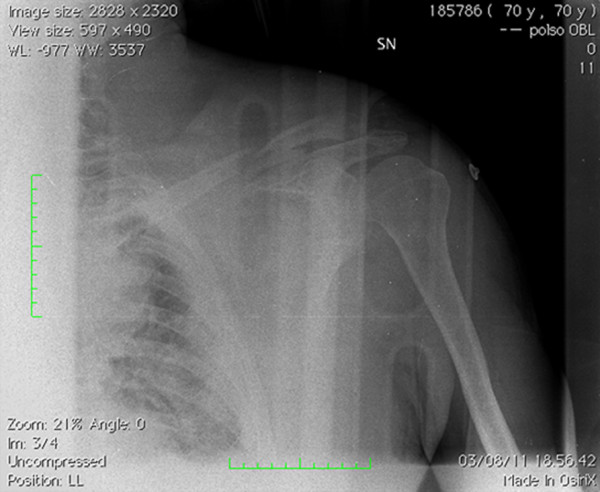
Plain radiography showing left midshaft clavicular fracture.

On admittance to our hospital the patient was conscious and well oriented, and physical examination revealed patient airways, no *cornage* nor *triage* were present, he was breathing normally, not complaining about dyspnoea, his respiratory rate was 20 per minute, the trachea was lying on the midline, there were no jugular veins turgor, vescicular murmur was bilaterally present and symmetric; a chest plain radiography was performed, there were no sign of pneumothorax but a left midishaft clavicular fracture was highlighted (Figure 1). The patient was hemodynamically stable, the skin was warm and dry, blood pressure was 120/90 mmHg with a 100 bpm heart rate, and he was resuscitated with 2000 ml of isotonic physiologic solution. He underwent a *Focused Assessment with Sonography for Trauma* (ECO-FAST), which showed no sign of active abdominal bleeding. There were no evidence of any neurological signs, his Glasgow Coma Scale (GCS) was 15, pupils were bilaterally isochoric, isocyclic, and reactive to light, and he was able to move the four limbs. The patient presented left parietal and periorbital ecchymotic excoriated contusion, as well as a vast hematoma with multiple excoriation in the left clavicular region and the left upper limb. The left hemithorax presented with multiple ecchymosis and was tender to palpation, while the right one was normal. There was no subcutaneous crepitation. The abdomen was flat, with physiologic respiration-associated mobility, there was no rebound tenderness, and peristalsis was present. The pelvis was stable. Palpable distal pulses were present in all extremities, and motor function of the lower limbs was preserved. Radial pulse of the left arm was slightly reduced and the limb presented with no evidence of neurological deficits (sensation, finger motility).

Urinary catheterization was performed, with an outcome of 100 ml of limpid urine. Laboratory tests showed an increase in myocytolysis enzymes with no evidence of cardiac failure (CPK = 569 UI/l; MB = 645.3 ng/ml; LDH = 338 UI/l). The haemoglobin value was initially 10.6 g/dl.

**Figure 2:**
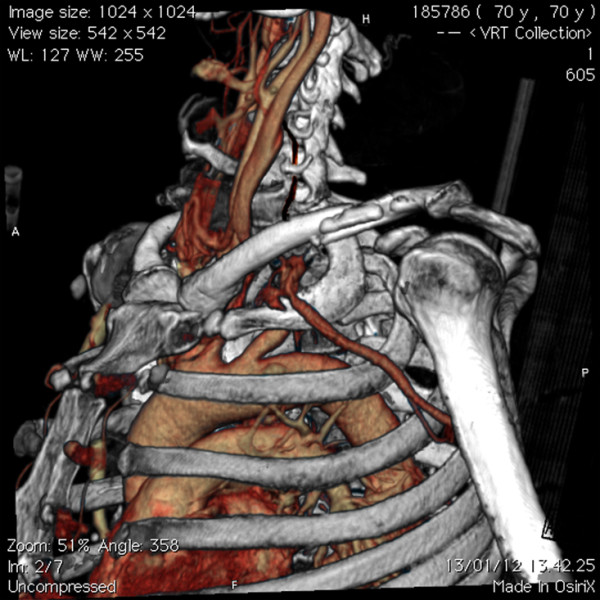
CT 3D reconstruction showing active left subclavian arterial bleeding and the left midshaft clavicular fracture.

The patient underwent to a total body CT scan. The CT showed left parietal bone fracture with no signs of intracranial haemorrhage, confirmed the left clavicualr fracture viewed at RX, and revealed active bleeding from left subclavian artery; a L1 vertebral soma fracture determining medulla compression was also detected, while the abdominal scans did not show any sign of visceral trauma (Figure 2).

Because of the subclavian active bleeding the patient was sent to interventional radiology operatory theatre.

**Figure 3:**
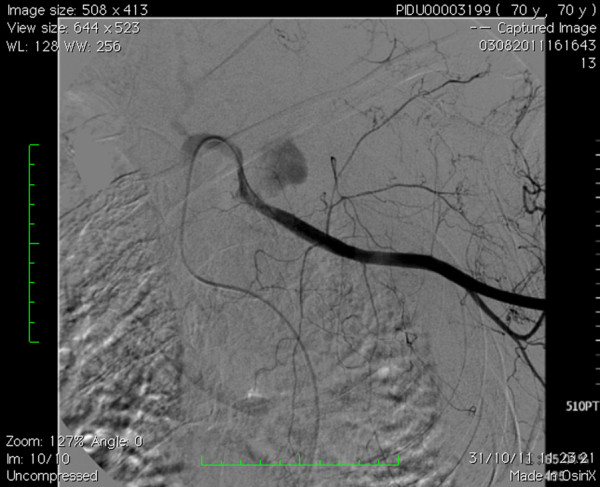
Arteriogram highlighting active left subclavian arterial bleeding, 3 cm after homolateral vertebral artery.

**Figure 4:**
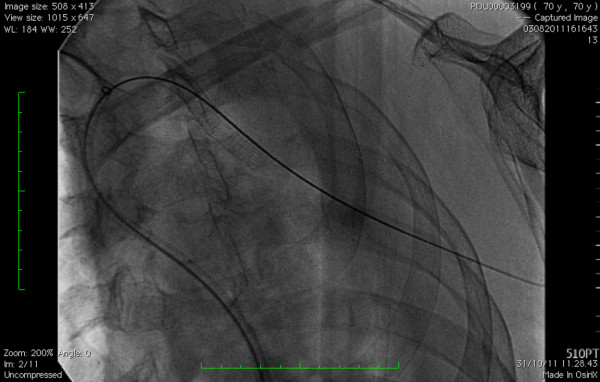
Covered Stent position.

**Figure 5:**
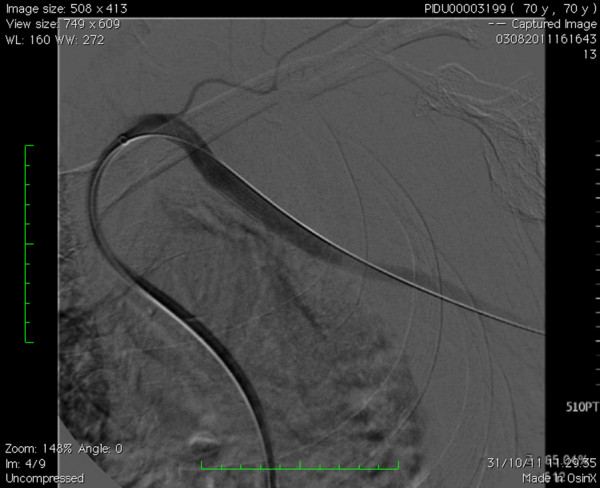
Arteriogram showing bleeding stop.

The right femoral artery was accessed using a standard Seldinger technique and a standard short 5F sheath was placed; a guidewire and a selective catheter were then used to cannulate the target vessel, and the left subclavian artery selective arteriography showed active bleeding from its 3^rd^ segment, 3 cm after the vertebral artery’s origin, due to a subtotal lesion of the arterial wall (Figure 3). A 8 × 50 mm Viabahn stent graft was advanced in anterograde fashion, then it was deployed under fluoroscopic visualization. An angioplasty balloon of appropriate size is used to iron out the proximal and distal edges of the stent and bring it up to profile (Figure 4). Next angiograms showed no active bleeding (Figure 5).

After surgical procedure, haemoglobin was checked again, and its value was 8.5 g/dl.

During the next days the patient underwent 2 blood transfusions, and its haemoglobin values returned between normal ranges (10.8 g/dl on the 6th day after trauma).

The L1 vertebral soma fracture was treated on the 9^th^ day after trauma.

The patient was discharged on the 15^th^ day after trauma.

## Discussion and review of literature

The association of subclavian arterial rupture and blunt thoracic trauma has been analyzed in many reports: in an article by Rulliat and coll. the incidence of subclavian arterial rupture among 1181 thoracic traumatic injuries was 0.4% [4]; a recent study by Shalhub and coll. reported a 47% incidence of subclavian arterial rupture above all the blunt thoracic outlet arterial injuries (BTOAI) [5]; furthermore, clavicular fractures were cited as the cause of 50% of traumatic subclavian artery injuries in another article by Kendall and coll. [1].

Subclavian artery injuries occurs from either elongation (stretching) or laceration mechanisms. Elongation is characteristically associated with a blunt force applied to the anterior shoulder or clavicle, as in motor vehicle crashes. This force is transmitted to fixed points along the vessel, typically the origin of the vertebral and internal thoracic artery where the vessel is then pulled apart. Laceration to the subclavian artery ensues from bony fragments produced by a fractured first rib or clavicle. The fracture is displaced into the vessel by the traction of associated chest wall muscles. Fractured clavicle has been cited as the cause of 50% of traumatic subclavian arterial injuries [1].

Subclavian arterial rupture is an uncommon complication of blunt thoracic trauma, and must be carefully ruled out because of its poor prognosis; in 1983 Sturm and Cicero have devised five criteria that should lead the examining physician to confirm the suspicion of arterial injury with arch aortography. These criteria include first rib fracture, diminished or absent radial pulses, palpable supraclavicular hematoma, chest roentgenogram demonstrating a widened mediastinum or hematoma over the area of the subclavian artery, and brachial plexus palsy [6]. Physical examination of the upper limb must focus on skin color, temperature, sensation, hand motility as well as radial pulse.

CT represents a key diagnostic exam, while selective arteriography offers both diagnostic accuracy and an operative approach.

Once identified, these injuries have historically been managed with a conventional surgical approach, associated with its own morbidity. Open repair is technically challenging and associated with significant morbidity and mortality for a variety of reasons. Exposure to obtain proximal control requires either a median sternotomy for the innominate and proximal right subclavian artery or a high anterolateral thoracotomy with potential clavicular resection for the proximal left subclavian artery. Such extensive incisions require lengthy healing and rehabilitation and carry significant morbidities.

Endovascular treatment represents a less invasive approach to these vascular injuries; furthermore, it offers less blood loss and a lesser invasive approach to an anatomically challenging problem [5]. Foremost is the benefit of approaching the lesion from a remote access site, which avoids major operative dissection in the traumatised area, and decrease the risk of injuring important surrounding structures, such as the subclavian vein or brachial plexus, which may be difficult to identify because of haemorrhage or involvement in the original injury [7]. High success rates can be achieved if the lesion is focal and can be traversed safely with a guidewire. Complete vessel transection has been reported as a common cause for failure of an endovascular approach, primarily due to difficulty with crossing the complete transection and its associated hematoma [8]. As such, vessel transection has traditionally been approached with open vascular reconstruction. It seems convenient to perform a femoral artery access in a trauma setting, for the possibility to perform selective arteriographies of abdominal viscera. Even though rare tortuosity of supra-aortic vessels could be an obstacle for catheterization, the femoral access offers the possibility to use devices of different dimensions (until 7 F), representing the standard access for this procedure. The brachial access still offers a valid alternative in case of difficult subclavian catheterization and provides the opportunity to perform a combined brachial and femoral access to create a through-and-through brachial-femoral wire and repair of transected mid-to-distal subclavian or axillary artery with covered stent, as described by Shalhub and coll. in their recent work [9].

Analyzing the past 24 years literature [Table 1], we found out 750 subclavian arterial lesions, reported in 12 different works (associated axillo-subclavian lesions where not included in our review). Among these series, 79 patients underwent endovascular repair (10.5%). Arterial injuries were caused by blunt trauma in 56 cases (7.5%), and endovascular repair was performed in 5 of these cases (8.9%).

**Table 1:** Past 24 years subclavian arterial injuries’ reports

**Year**	**Authors**	**Number of cases**	**Blut trauma**	**Penetrating trauma**	**Endovascular repair**	
					**Blunt**	**Penetreting**
1988	Costa et al.	167	15	152	0	0
1996	Patel et al.	6^a^	-	6	-	6
1999	Cox et al.	56	25	31	0	0
1999	Demetriades et al.	79^a^	-	79	-	1
1999	Janne d’Othée et al.	1^b,c^	1	-	1	-
2000	McKinley et al.	260	11	249	0	0
2003	Lin et al.	54^a^	-	54	-	0
2005	Castelli et al.	4^c^	1	3	1	3
2005	Bukhari et al.	1^b,c^	1	-	1	-
2008	du Toit et al.	57^a,c^	-	57	-	57
2009	Sobnach et al.	50^a^	-	50	-	1
2010	Carrick et al.	15	2	13	2	6

a - This report enrolls only Penetrating Arterial Injuries.

b - This is a Case Report.

c - This report analyses only Endovascular Treatments.

This review highlights the rarity of endovascular approach to subclavian arterial injuries: on the overall 569 cases reported from 1988 to 2000, only 8 (1.4%) underwent endovascular treatment; on the other hand, in the past 12 years 71 (39.2%) of 181 cases reported in literature were treated by endovascular approach [7,10-20].

Our analysis points out how the technical progresses and growing experience of vascular surgeons has improved the feasibility of endovascular treatment, creating a valid alternative to challenging ‘classic’ surgical approaches.

## Conclusions

Our analysis reveals a continuous growth in the application of endovascular stent grafting vs. open repair for subclavian arterial injuries, thanks to the growing experience of endovascular surgeons coupled to rapid technologies’ development. Furthermore, the indications for endovascular stent grafting were stretched: in 2005, hemodynamical instability status was still pointed out as a contraindication to endovascular approach, as well as complete vessel transaction [21]; 6 years later, the series by Shalhub and coll. [5] extended the indication to hemodynamically unstable patients as well as to patients reporting complete vessel transaction thanks to the application of a new endovascular technique based on the use of a combined brachial and femoral arterial access to create a brachial-femoral wire and repair of transected mid-to-distal subclavian or axillary artery [9]. In our opinion, according to the observation by Danetz [21]and Shalhub [5], the creation of an OR environment with full endovascular capability, where open and endovascular techniques can be used as well as other necessary procedures such as exploratory laparotomy and orthopedic fixation, without the need to transport the unstable patient, is crucial for a fast and multidisciplinary management of trauma patients.

## Consent

Written informed consent was obtained from the patient for publication of this case report and any accompanying images. A copy of the written consent is available for review by the editor-in-chief of this journal.

## Competing interests

The authors declare that they have no competing interests.

## Authors’ contributions

MA coordinated the whole team work. LC, GC, LV cared about bibliographical research, images’ collection and first draft writing. MC reviewed the radiological aspects of the article. CM carried out the final internal review. All authors read and approved the final manuscript.
